# Family Planning Prevalence Among Postpartum Mothers Attending Child Welfare Clinics – A Sri Lankan Experience

**DOI:** 10.4103/0970-0218.55300

**Published:** 2009-07

**Authors:** Suneth B Agampodi, Thilini C Agampodi, Pushpika Chandrasekara

**Affiliations:** Medical Officers of Health (Beruwala), Purwarama Road, Aluthgama, Sri Lanka. E-mail: sunethagampodi@yahoo.com

Sir,

One of the main reasons for seeking abortion in Sri Lanka is insufficient spacing between births.(1) This could be partly due to deficit in postpartum contraceptive services. A hospital-based study reported that at the time they leave the hospital, knowledge on the appropriate time to start a contraceptive method was poor among Sri Lankan mothers.(2) Even among those who are knowledgeable, the actual practices are unknown. Studies have demonstrated that antenatal programs that promote contraception are not effective to improve postpartum contraceptive prevalence.(3,4) The purpose of this study was to assess the prevalence of modern Family Planning (FP) methods among postpartum mothers in order to improve maternal and child healthcare service provision.

A clinic-based, cross-sectional, descriptive study was conducted in the area of the medical officer of health, Beruwala. We interviewed all infant-mother pairs where infants were two months of age, attending child welfare clinics for the first dose of Diphtheria-Tetanus-Pertussis (DTP) during a period of two months. A cluster sampling technique was used to obtain the sample from 19 child welfare clinics. An interviewer administered questionnaire was used for data collection. Authors collected data during routine clinic visits. Ethical and administrative clearance was obtained from the National Institute of Health Sciences, Kalutara.

All together 129 mothers were interviewed. Mean age of the study sample was 27 years with a standard deviation of 4.6. Median duration of the postpartum period was eight weeks. Sixty-four of the study sample were having their first child. The sample consisted of 58 (45%) Muslim mothers and 71 (55%) Sinhalese mothers.

Family Planning (FP) prevalence among study participants was 41.1% (53). Out of the mothers who were not on FP, 22 (28.9%) had already decided on an FP method. Fifty-four mothers, 41.9%, neither used a family planning method nor had they decided on a method or when to start. All these 75 mothers who had already started and decided on an FP method had received domiciliary care (postpartum home visits by the public health midwife) and FP advice through the area public health midwife.

Among those who had not decided on a method, 40 (74.1%) had no specific reasons for noncommencement, while 12 (22.2%) had not decided because of the inaccurate information received on FP. Nine mothers (16.7%) did not receive domiciliary care at all and only 33 (61.1%) received family planning advice from the area public health midwife.

Use of an FP method was significantly associated with domiciliary postpartum care (Fishers exact test *P* = 0.009) and FP counseling (χ^2^ = 17.5, *P* < 0.001). Maternal age, ethnicity or number of children was not significantly associated with postpartum FP prevalence.

The prevalence of modern methods used among the reproductive age group in Sri Lanka was 49.5% in 2000.(5) The prevalence of modern FP methods used among postpartum mothers in our study sample was below this national average. The study shows that all mothers who received domiciliary care had not received FP counseling. Divisional level health care managers should pay more attention to improve the quality of postpartum care in order to improve maternal and child health status.
